# SARS-CoV-2 mutations: the biological trackway towards viral fitness

**DOI:** 10.1017/S0950268821001060

**Published:** 2021-04-30

**Authors:** Parinita Majumdar, Sougata Niyogi

**Affiliations:** 1 Independent Researcher, Kolkata, India; 2 Dinabandhu Andrews Institute of Technology and Management, Block-S, 1/406A, Patuli, Kolkata, West Bengal 700094, India

**Keywords:** Fitness, Mutation, SARS-CoV-2, Transmission, Virulence

## Abstract

The outbreak of pneumonia-like respiratory disorder at China and its rapid transmission world-wide resulted in public health emergency, which brought lineage B betacoronaviridae SARS-CoV-2 (severe acute respiratory syndrome coronavirus 2) into spotlight. The fairly high mutation rate, frequent recombination and interspecies transmission in betacoronaviridae are largely responsible for their temporal changes in infectivity and virulence. Investigation of global SARS-CoV-2 genotypes revealed considerable mutations in structural, non-structural, accessory proteins as well as untranslated regions. Among the various types of mutations, single-nucleotide substitutions are the predominant ones. In addition, insertion, deletion and frame-shift mutations are also reported, albeit at a lower frequency. Among the structural proteins, spike glycoprotein and nucleocapsid phosphoprotein accumulated a larger number of mutations whereas envelope and membrane proteins are mostly conserved. Spike protein and RNA-dependent RNA polymerase variants, D614G and P323L in combination became dominant world-wide. Divergent genetic variants created serious challenge towards the development of therapeutics and vaccines. This review will consolidate mutations in different SARS-CoV-2 proteins and their implications on viral fitness.

## Introduction

The emergence of pneumonia with unknown aetiology at Wuhan province of China in December 2019, eventually led to the identification of a novel strain of human coronavirus (CoV) named severe acute respiratory syndrome coronavirus 2 (SARS-CoV-2) based on its genetic relatedness with SARS-CoV, the causative agent of severe acute respiratory syndrome outbreak in 2002 [1–4]. High transmission dynamics and overwhelming infection rate of SARS-CoV-2 resulted in declaration of COVID-19 (*Co*rona*vi*rus *D*isease 2019) pandemic on 11th March 2020 by the World Health Organization (WHO) (https://www.who.int). The infectivity of SARS-CoV-2 is distinctly higher among the members of betacoronaviridae with a comparatively lower case fatality rate (CFR) of 1.4–2.1% compared to SARS-CoV (9.6%) and MERS-CoV (middle east respiratory syndrome coronavirus) (40%) [5, 6]. Several studies had highlighted the association of different lockdown strategies, viral testing capabilities and varied demographic compositions with the severity of COVID-19 pandemic [7–10]. Since 1960s with the discovery of first human CoV until date, altogether seven human CoVs are identified [1, 3, 4]. Among these seven strains, SARS-CoV, MERS-CoV and SARS-CoV-2 are associated with acute human respiratory disorder whereas the remaining four strains 229E, OC43, NL63 and HKU1 showed mild clinical symptoms including sore throat, nasal discharge, fever and cough [2, 11]. The average mutation rate of 4 × 10^−4^ nucleotide substitutions/site/year is largely, if not exclusively, responsible for the genetic diversity of betacoronaviridae [12]. In addition to mutation, frequent recombination and interspecies transmission are also common among them [11]. These factors largely account for temporal change in their infectivity and virulence. Recent studies highlighted the implication of mutations in the rapid community transmission of SARS-CoV-2- and COVID-19-associated mortality [13]. In order to understand the evolutionary trend in SARS-CoV-2, it is of utmost importance to study the mutation patterns and their effect on viral fitness. The current review aims to provide a comprehensive knowledge on SARS-CoV-2 mutations and their impact on the major viral proteins associated with viral life-cycle, pathogenicity and virulence.

## Genome organisation of SARS-CoV-2

**Fig. 1.**
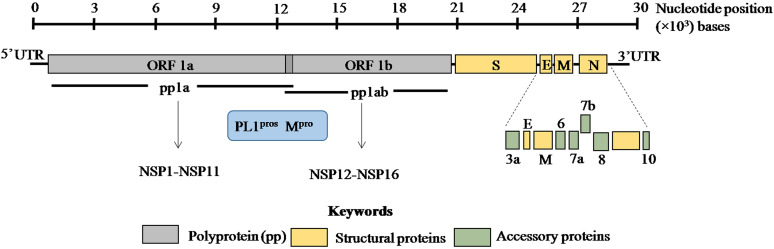
ORF1a and ORF1b encode two overlapping poly-proteins pp1a and pp1ab which are proteolytically processed into 16 non-structural proteins (NSP1–NSP16) by the main protease (M^pro^) and papain-like proteases (PL1^pros^). The scale bar on the top denotes the nucleotide position of the genome.

The viral genome is non-segmented, single-stranded positive sense RNA, ~30 kb in size with 5′ and 3′ untranslated regions (UTRs) (Fig. 1) [1, 2, 14]. Genome analysis of SARS-CoV-2 revealed 79% and 50% identity with SARS-CoV and MERS-CoV, respectively [1, 2]. Moreover, 88% homology was observed with two bat coronaviruses, bat-SL-CoVZC45 and bat-SL-CoVZXC21 suggesting a plausible bat origin of SARS-CoV-2.

SARS-CoV-2 genome encodes ORF1a/ORF1ab (open reading frame) polyproteins and four structural proteins including S (spike), E (envelope), M (membrane) and N (nucleocapsid) with several intervening ORFs encoding accessory proteins [2, 14] (Fig. 1). Among these ORFs, ORF1a and ORF1b at the 5′ terminus comprise 2/3rd of the genome and encode two overlapping poly-proteins pp1a and pp1ab [1–3] (Fig. 1). These poly-proteins undergo proteolytic cleavage by the viral main protease (M^pro^) which has at least 11 conserved cleavage sites and papain-like proteases (PL^pros^) to generate 16 non-structural proteins (Fig. 1) [15, 16]. These non-structural proteins have multi-faceted role in viral replication, transcription, morphogenesis as well as evasion of host immune response. On the contrary, accessory proteins are not crucial for viral life cycle but play important role in viral pathogenesis [17]. The biological functions of these structural, non-structural and accessory proteins in SARS-CoV-2 are discussed in Table 1.

**Table 1.** Functions of various SARS-CoV-2 proteins

Non-structural proteins	Functions	Reference
NSP1	Interacts with 40S ribosome and inhibits host translation. Degrades host mRNA and facilitates viral gene expression. Evasion of host immune response	18, 19
NSP2	Viral replication	20, 21
NSP3	Proteolytic cleavage of replicase poly-protein at its N terminus. Participates in viral replication by assembly of cytoplasmic double membrane vesicle. De-ubiquitinates cellular proteins tagged with Lys48 and Lys63-linked poly-ubiquitin chain. Type I interferon mediated immune response antagonist. Blocks NF-kappa-*β* signal transduction	16, 20, 22
NSP4	Assembly of cytoplasmic double membrane vesicle and helps in viral replication	23, 24
NSP5	Proteolytic cleavage of replicase poly-protein at its C terminus	15
NSP6	Triggers autophagosome formation from host endoplasmic reticulum	Swiss-model repository (https://swissmodel.expasy.org/) 25
NSP7	Cofactor ofRdRp	16, 26
NSP8	Cofactor of RdRp	16, 26
NSP9	Binds single-stranded RNA and participates in viral replication	27
NSP10	Methylates the 5′ cap structure of viral mRNA	28, 29
NSP11	Not identified	
NSP12	Replication and transcription	26
NSP13	Helicase, nucleoside triphosphatase, have 5′ RNA triphosphatase activity and potent interferon antagonising activity	30–32
NSP14	Cleaves single-stranded and double-stranded RNA from 3′ to 5′ end and has N7-guanine methyl-transferase activity. Exoribonuclease activity, interferon antagonising activity	32–34
NSP15	Harbours endo-ribonuclease activity, interferon antagonising activity	32, 35
NSP16	Possesses nucleoside-2′ *O*-methyl-transferase activity	36
Structural proteins		
S	Binds to ACE2 host cell receptor and mediates viral entry within the host cell	2, 37
E	Maturation of virion, forms viroporin on host membrane and facilitate ion transport	38
M	Maintains spherical membrane curvature of the virus, stabilises nucleocapsid and facilitates viral assembly, antagonises type I and III interferon responses	39
N	Encapsidates viral nucleic acid	40
Accessory proteins		
ORF3a	Induces apoptosis, helps viral entry, blocks STAT1 and inhibits IFN activity	41, 42
ORF6	Antagonises interferon signalling by blocking nuclear entry of STAT1 via Rae1 and Nup98	43
ORF8	Immune evasion by down-regulating the surface expression of MHC I	44
ORF10	Ubiquitin ligase, interacts with CUL2 and degrades host proteins	45
ORF7a	Interacts with CD14+ monocytes and triggers aberrant inflammatory responses, inhibits STAT2 and antagonises IFN	46
ORF7b	Inhibits both STAT1, STAT2 and blocks IFN stimulated gene expression	42

### Mutations in SARS-CoV-2 genome

Since its emergence in 2019, SARS-CoV-2 infection had become widespread with 126 210 104 confirmed cases in more than 200 countries with a death toll of 2 769 638 as on 26th March 2021 (https://www.who.int). Following the sequencing of SARS-CoV-2 genome at Wuhan in December 2019, more than 10 000 genetic variants are reported [8–10]. Recently an emergent variant of SARS-CoV-2, VUI202012/01 (variant under investigation, year 2020, month December, variant 01) or VOC202012/01 (variant of concern) or B.1.1.7 in the United Kingdom with an enhanced transmissibility of 56–70% became a major concern [47, 48]. The variant strain with 14 non-synonymous mutations and three deletions transcend the existing variants at London, East and South East England [47]. The rapid spread of COVID-19 among individuals of different ages, genetic compositions and medical predispositions provides suitable mutagenic backdrop for generation of heterogeneous SARS-CoV-2 population.

### Predominant mutation clusters in SARS-CoV-2 genome

An average of ⩾11 mutations per sample with the insurgence of single-nucleotide substitutions was reported for SARS-CoV-2 [8, 49]. These mutations are categorised as amino acid changing SNP (single-nucleotide polymorphism), amino acid changing triplet, 5′ UTR-SNP and silent SNP. Notably, C → T (55.1%) transition was more common than A → G (14.8%) transition and G → T transversion had an occurrence of 12%. SNP variants are classified into six clusters based on the pattern of co-mutation [10]. Cluster I includes 3037C>T; NSP3:F106F (non-structural protein3:F106F) and 14408C>T; RdRp:P323L, cluster II includes 3037C>T, 14408C>T and 23403A>G; S:D614G, cluster III includes 14408C>T, cluster IV includes 3037C>T, 14408C>T, 23403A>G, 28881G>A; N:R203K, 28882G>A; N:R203K, 28883G>C; N:G204R, cluster V includes 3037C>T, 14408C>T, 23403A>G and 25563G>T; ORF3a:Q57H and cluster VI includes 8782C>T; NSP4:S76S, 28144T>C; ORF8:L84S [8, 10]. Among these six clusters, clusters III, IV and VI were predominant in Asian countries whereas clusters IV, V and VI were prevalent in the United States. In addition to SNPs, in-frame deletions and short frame-shift deletions were also observed among the genetic variants with a very low frequency of 0.6% and 0.8% respectively. However, insertion mutation was extremely rare with <0.1% among all the mutations [10].

Based on the specific mutation patterns, the genetic variants of SARS-CoV-2 are classified into three major phylogenetic clades: G, S and V. The clade G, S and V comprise variants of S:D614G (23403A>G), ORF8:L84S (8782C>T) and ORF3a:G251V (26144G>T), respectively [8] (Table 2). Clade G and V variants comprise amino acid changing SNPs whereas clade S variant include silent SNP. Clade G has two offspring, GH and GR based on the emergence of nascent mutations, in addition to the already existing one. GR clade has a combination of spike D614G and nucleocapsid RG203KR mutations, prevalent in Europe and South America while GH comprises mutations in spike D614G and ORF3a Q57H which predominates in North America.

**Table 2.** Different mutations in SARS-CoV-2 proteins

Protein name	Non-synonymous amino acid mutations	Reference
Spike protein	P323L, A97V, T141I, A449V, D63Y, Q239K, V341I, A435S, K458R, I472V, H519P, A831V, S943T, N439K, L452R, A475V, V483A, F490L, Y508H, V1176F, S4777N, F32I, H49Y, S247R, N354D D614G + V341I, D614G + K458R, D614G + I472V, D614G + A435S N501Y, P681H, A570D, T716I, S982A, D1118H, Δ69/Δ70, Δ144/145	10, 47, 50–54
Nucleocapsid protein	R203K, G204R, P13L, S188L, S202N, D103Y, I292T, S194L, S197L, T339I, T148I, P344S	10, 54, 55
Membrane protein	T175M, D3G, C64Y, S4F, R158C, I52T, I76F, T7I, F193L, G78C	10, 56
Envelop protein	T9I, V24M, V58F, L73F	10, 56
RdRp	P323L, A97V, T141I, A449V, D63Y	10, 57
ORF3a	G251V, W128L, L127I, Q57H, W131C, L129F, D173Y, H93Y, P25L, T175I, L94F, K16N, W149L	56, 58
ORF6	P57L, T21I	56
ORF7a	E92D, M1R, L5F, A8S, Y20N, R78H, A105S, A106S	56
ORF8	Q91K, Q72H, P36S, I9T, P30S, R52T, E106Q, A65V, F120L, I121L, R101L, G66S, Q72H, L84S	56, 59
ORF10	D31Y	56

### Mutation in RNA-dependent RNA polymerase

Variants of RNA-dependent RNA polymerase (RdRp) emerged early during the COVID-19 outbreak in Europe, North America, China and Asian countries and hence was considered as a mutation hotspot [10, 57]. A total of 607 mutations are reported in RdRp of which 14408C>T (P323L) mutation which lies near the interface domain of RdRp showed highest frequency (10 925 times in 15 140 genotypes) [10] (Table 2). This variant of RdRp did not alter the catalytic activity but is likely to abrogate the interaction with its cofactors and existing anti-viral drugs [57]. Crystal structure analysis revealed that RdRp (NSP12) forms a complex with NSP7 and NSP8 which provide processivity to the polymerase [26]. However, specific residues involved in their interaction remain unresolved. Unlike RNA viruses, RdRp of CoVs has proof reading activity, a characteristic of *Nidovirales*, which is conferred by 3′ → 5′ exonuclease ExoN/NSP14 [60]. An *in vitro* biochemical assays could detect interactions between NSP12-NSP7-NSP8 and ExoN/NSP14. Such an interaction is necessary for the excision of wrongly incorporated bases from nascent RNA.

The 14408C>T (P323L) mutation was found to be associated with increasing point mutations in viral isolates in Europe during the early phase of COVID-19 outbreak. Thus, it is possible that mutations in RdRp might alter the interaction of RdRp with these cofactors which could render the proofreading activity less effective leading to the emergence of numerous SARS-CoV-2 variants [57]. *In silico* analysis predicted the docking site of anti-viral drugs within a hydrophobic cleft located near the 14408C>T mutation site [57]. This mutation was predicted to diminish the affinity of RdRp for existing anti-viral drugs. Mutation in the catalytic domain of RdRp, D484Y resulted in remdesivir resistance, the first anti-viral drug used in the United States [61]. Thus, the emergence of RdRp genetic variants in SARS-CoV-2 posed tremendous challenge towards the efficacy of anti-viral therapeutics.

### Mutation in spike protein

Spike glycoprotein mediates viral entry within the host cell by interacting with the membrane-bound angiotensin-converting enzyme 2 (ACE2) and plays a remarkable role in SARS-CoV-2 infectivity and transmissibility [37, 62]. A 1273 amino acid containing spike protein can be divided into S1 and S2 subunits [63]. The C terminal domain of S1 in SARS-CoV-2 harbours the receptor binding domain (RBD) and the residues 442–487 are crucial for interaction with the host cell receptor [62]. S2 subunit is crucial for mediating host–viral membrane fusion [37, 63]. Mutations are continuously being reported for S gene having 1004 unique mutations among 15 140 genotypes and found out to be the second most non-conserved protein in SARS-CoV-2 after nucleocapsid protein [10]. Notably, mutations are more frequent in S1 unit and in past few months, almost half of the amino acid residues in RBD had been mutated creating a major challenge for vaccine development. Mutations in S protein have multiple consequences including altered protein stability, receptor affinity and sensitivity to neutralising monoclonal antibody (mAb) as well as convalescent serum [50, 51, 64]. R408I mutation stabilising S protein was reported in an Indian strain [64]. Among all S protein variants, D614G increased at an alarming rate which was observed 10 969 times in 15 140 genome isolates, suggesting a positive selection of this variant during the course of viral evolution [10]. D614G variant was highly transmissible and became predominant in Europe, Canada, Australia and United States [65]. Moreover, this particular variant of SARS-CoV-2 was more infectious and found to be associated with enhanced mortality across the world [13]. Structural analysis revealed D614G mutation favours open conformation of S protein which facilitates binding with the host receptor thereby enhances its infectivity [66]. Two new variants, V1176F and S4777N are also associated with higher mortality and found to spread rapidly across the world [50]. V1176F arose independently and also co-occurred with D614G. *In silico* analysis predicts V1176F variant could facilitate the interaction with ACE2 by stabilising spike protein trimeric complex. The co-mutations D614G + V341I, D614G + K458R and D614G + I472V fall within the RBD of S protein and enhance the infectivity of virus by favouring binding with the host receptor [51].

VUI202012/01 had eight mutations in S protein of which N501Y, P681H, Δ69 and Δ70 have potential implications on viral infectivity [47, 52]. N501 is one of the six key residues mediating contact with the host cell receptor [37]. N501Y falls within the RBD and had been shown to enhance the binding affinity of S protein with human ACE2 [47]. Deletion of two amino acids at positions 69 and 70 of S protein is likely to be associated with host immune evasion and increased infectivity [52]. The furin cleavage site near S1/S2 is a unique feature of SARS-CoV-2 and is linked with viral infectivity [63]. P681H mutation lies near the furin cleavage site and might interfere with viral infectivity and transmission [47]. In addition to these mutations, A570D (RBD), Δ144/145 (S1 subunit), T716I, S982A and D1118H (S2 subunit) are also reported in VUI202012/01 [52]. The precise role of these mutations in viral life cycle and pathogenesis is currently under investigation.

The S2 unit comprises of fusion peptide (FP), heptad repeat 1 (HR1), HR2, transmembrane domain and cytoplasmic domain [63]. The insertion of four amino acids upstream of HR1 at positions 681–684 increases the length and flexibility of the connecting region between the FP and HR1 [67]. This favours viral entry within the host and also serves as a genetic determinant of SARS-CoV-2 pathogenicity. Several mutations including A475V, N439K, L452R, F490L, V483A and Y508H in S protein resulted in decreased sensitivity to mAb [51–53, 65–67]. The antigenic properties of S protein had already been exploited in vaccine development. Thus, it is crucial to understand the evolution of S protein antigenicity by studying their mutation patterns and subsequent implications on viral pathogenesis.

### Genetic determinant of SARS-CoV-2 virulence and N protein mutation

Nucleocapsid phosphoprotein has multi-faceted role in SARS-CoV-2 life cycle including replication of viral genome, assembly of mature virions and encapsidation of viral nucleic acid [68]. The positively charged amino acid residues in the N terminal domain of nucleocapsid protein (46–176 amino acids) and serine/arginine-rich linker region (184–204 amino acids) are important for interaction with viral RNA [69, 70]. The C terminal dimerisation domain also facilitates RNA binding. Moreover, N protein helps to unwind viral RNA following infection through phosphorylation of specific amino acid residues involved in such RNA–protein interaction. Any mutation affecting the phosphorylation sites of N protein is likely to interfere with viral life cycle. R203K, G204R, P13L, D128D, L139L, S188L, S202N, D103Y and I292T mutations are more frequently observed in N protein [10] (Table 2). However, the biological implications of these mutations warrant further investigation.

An enrichment of positively charged amino acid within the NLS (nuclear localisation signal) of nucleocapsid proteins compared to the less harmful CoVs including HKU1, NL63, OC43 and 229E is considered as one of the genetic determinants of SARS-CoV-2 pathogenicity [67]. Such enrichment is also present in SARS-CoV and MERS-CoV nucleocapsid proteins indicating convergent evolution. The abundance of positively charged residues is expected to strengthen the nuclear localisation of N protein and thereby facilitates its interaction with viral as well as host proteins [67]. Thus, mutations strengthening the NLS of N protein could affect its subcellular localisation and subsequent interaction with host proteins.

### Co-mutations in SARS-CoV-2

SARS-CoV-2 variants with certain co-mutations became prevalent world-wide compared to single mutation suggesting their fitness [66]. NSP3:F106F (3037C>T) mutation co-evolved with RdRp:P323L, S:D614G, N:R203K, N:G204R and ORF3a:Q57H mutations and these strains with co-mutations were predominant in Russia, United States and Europe [10, 71]. Since 3037C>T mutation is silent and does not have major impact on NSP3 protein *per se*, it may change codon usage and thereby might affect the translation efficiency of NSP3 [8]. Mutations in NSP3 had been linked with positive selection of viruses leading to evolution in betacoronaviruses [72]. Interestingly, 3037C>T, 14408C>T and 23403A>G co-mutations had the highest number of descendants world-wide indicating positive selection of this epidemiologically dominant SARS-CoV-2 variants. In addition to this co-mutation, a novel non-synonymous mutation NSP3:S1515F (4809C>T) was observed only in Indian strains early in March 2020 [71]. NSP3 interacts with nucleocapsid protein and tethers the nascently translated replicase–transcriptase complex to the viral genome during the early stages of infection in SARS-CoV [73]. *In silico* analysis predicts this mutation as a stabilising one and it is intriguing to address whether this mutation strengthens the interaction of N protein with the replicase–transcriptase complex favouring viral infection.

### Mutations in accessory proteins

**Fig. 2.**
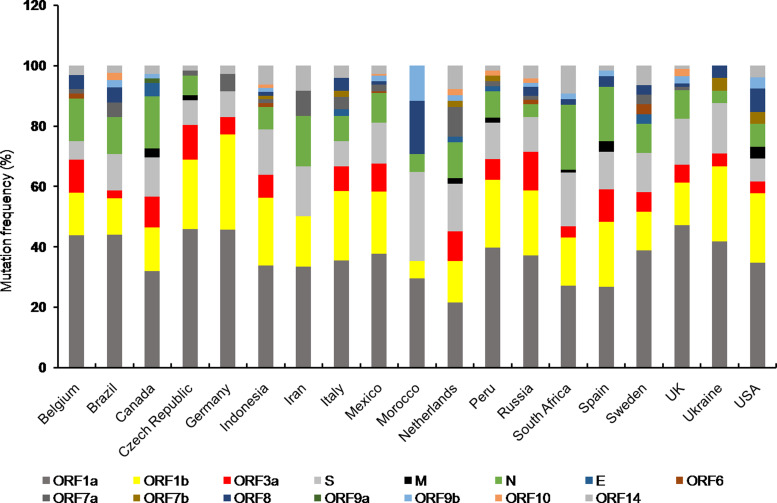
Stacked bar chart shows frequency distribution of mutations at various SARS-CoV-2 ORFs from indicated countries as of 29th December 2020. Mutations in SARS-CoV-2 proteins for respective countries were obtained from NextStrain open source project (https://nextstrain.org/ncov). Mutation frequency was calculated by dividing the number of mutations for a particular protein with total number of mutations corresponding to all the proteins for a given country, multiplied by 100.

Mutations are found in all the accessory proteins of SARS-CoV-2 with varying frequency (Fig. 2). Among the accessory proteins, ORF3a and ORF8 are brought into limelight due to the rapid spread of cluster V (NSP3:F106F, RdRp:P323L, S:D614G and ORF3a:Q57H) and VI (NSP4:S76S and ORF8:L84S) [10]. Mutation in ORF3a was associated with a higher CFR in the COVID-19 pandemic [56]. Among 51 non-synonymous mutations in ORF3a, Q57H (17.4%) and G251V (9.7%) were predominant ones [58] of which Q57H mutation was found to cause disease severity in hospitalised [74]. Moreover, Q57H mutation co-occurred with either of W131C, L129F and D173Y second site mutations [58]. ORF3a is the largest accessory protein (~30 kDa) in SARS-CoV-2 which elicits host inflammatory responses through activating innate immune receptor NLRP3 (NOD, LRR and pyrin domain containing 3) inflammasome [75]. This results in uncontrolled release of pro-inflammatory cytokines and other inflammatory mediators including tumour necrosis factor, interleukin-6, leukotrienes and prostaglandins, leading to cytokine storm, the clinical characteristic of SARS-CoV-2 pathogenesis [75, 76]. Mutations in ORF3a are predicted to cause loss of B cell epitopes thereby affects antigenicity of ORF3a [56]. Since ORF3a was predicted to interact with the host signalling pathways including JAK- STAT, chemokine and cytokine-related pathways, it is possible that ORF3a variants could aggravate host immune response leading to the varied severity of COVID-19 among infected individuals.

ORF8 is most divergent in SARS-CoV-2 with no paralogues or orthologues outside lineage B betacoronaviruses [59]. This suggests that ORF8 might play an important role in lineage specific adaptation of betacoronaviruses within the host [17]. SARS-CoV-2 ORF8 down-regulates MHCI expression on the surface of antigen-presenting cells which facilitates viral infection by evasion of host immune response [44, 77]. Mutational analysis revealed ORF8 locus is subjected to point mutations, non-sense mutation generating stop codon and deletion mutations [59]. Among the point mutations, L84S is the predominant one and associated with mild disease symptoms among the hospitalised individuals [59, 74]. Three deletion mutations of ORF8 are reported world-wide of which 382 nucleotide deletions resulted in complete loss of ORF8 and the terminal part of ORF7b. This variant was originated in Wuhan and traced to Taiwan and Singapore [59]. Notably, deletion of this locus was associated with milder infection due to reduced systemic release of cytokines and a better immune response to SARS-CoV-2 [78]. In addition to deletions, several non-synonymous amino acid substitutions in ORF8 are reported world-wide indicating positive natural selection of those variants [50].

A 27 amino acid in-frame deletion is reported for ORF7a locus [46]. Structural analysis revealed loss of putative signal peptide and first two beta strands from ORF7a, the orthologue of SARS-CoV ORF7a. However, the implication of such mutation on viral fitness needs further investigation.

## Conclusion

The unusually larger genome of CoVs among RNA viruses is primarily responsible for their daunting genome plasticity due to frequent mutation and recombination [1]. In addition to this, presence of error prone replication machinery in RNA viruses largely contributes to their genetic diversity with varying outcomes including shift in their biological properties, interspecies transmission and altered transmissibility [11, 79]. The overall outcome of mutations is reflected at the species level either by making it stronger or weaker. Any mutation which provides survival advantage is positively selected by nature and thus mutational studies are essential to understand the evolutionary trend at the organismal level [80]. Frequency distribution of mutations in different proteins of SARS-CoV-2 variants from countries with total infection >2 lakhs showed almost all the protein coding ORFs harboured mutations to a varying extent (Fig. 2). Furthermore, mutations in ORF1a, ORF1b, N and S proteins were present in almost all the countries of which Canada, South Africa and Spain showed comparatively higher number of mutations in N protein. However, Morocco had highest number of S protein mutations (Fig. 2).

Among the structural proteins, M and E had least number of variants indicating these are conserved proteins [10] (Fig. 2). The emergence of numerous genetic variants has brought SARS-CoV-2 into spotlight due to its enhanced transmissibility and infectivity compared to the original Wuhan strain [13]. Moreover, mutations in structural (spike) and accessory proteins (ORF3a) of SARS-CoV-2 are associated with a higher CFR of COVID-19 pandemic [13, 56, 65]. The nucleocapsid phosphoprotein and spike glycoprotein are among the most non-conserved proteins in SARS-CoV-2 posing a major challenge towards vaccine development [10]. Moreover, S protein variants are highly infectious due to effective binding with the host cell receptor. On the contrary, the other structural proteins including membrane and envelope were relatively more conserved suggesting perturbation within these genes are not encouraged which otherwise might affect viral integrity and life cycle [10]. Among the SARS-CoV-2 non-structural protein variants, deletion at position Asp268 of NSP2 spread rapidly in Europe [81]. Deletion of three amino acids, KSF towards the 3′ end of NSP1 at positions 241–243 was found in viral isolates from different geographical locations, suggesting their rapid spread [82]. Whether such mutations have any effect on viral pathogenicity needs to be explored.

There had been considerable advancements in the field of vaccines, therapeutic antibodies and anti-viral therapy to combat COVID-19 [51, 61]. However, the emergent genetic variants might undermine the effectiveness of those therapeutic interventions. With the outbreak of COVID-19 pandemic, there has been an explosive deposition of SARS-CoV-2 genome sequences in the repositories which made detailed analysis of SARS-CoV-2 genetic variants much easier. As COVID-19 pandemic progresses, closer investigation of those evolving strains of SARS-CoV-2 is crucial to understand the biological significance of the mutations on viral fitness.

## Data Availability

The data presented in this review paper would be available from the corresponding author upon request. The mutation data on SARS-CoV-2 variants are freely accessible from NextStrain open source project (https://nextstrain.org/ncov).
